# Soil gross nitrogen transformations along the Northeast China Transect (NECT) and their response to simulated rainfall events

**DOI:** 10.1038/srep22830

**Published:** 2016-03-07

**Authors:** Jin-bo Zhang, Liang Wang, Wei Zhao, Hui-feng Hu, Xiao-juan Feng, Christoph Müller, Zu-cong Cai

**Affiliations:** 1 School of Geography Sciences, Nanjing Normal University, Nanjing 210023, China; 2 Jiangsu Center for Collaborative Innovation in Geographical Information Resource Development and Application, Nanjing 210023, China; 3 Key Laboratory of Vitual Geographical Environment (VGE), Ministry of Education, Nanjing Normal University, Nanjing 210023, China; 4 Jiangsu Provincial Key Laboratory of Materials Cycling and Pollution Control, Nanjing 210023, China; 5 Institute of Botany, the Chinese Academy of Sciences, Beijing 100093, China; 6 Department of Plant Ecology (IFZ), Justus-Liebig University Giessen, Heinrich-Buff-Ring 26, 35392 Giessen, Germany; 7 School of Biology and Environmental Science, University College Dublin, Belfield, Dublin 4, Republic of Ireland.

## Abstract

Climate changes are predicted to increase extreme rainfall events in semiarid and arid region in Northern Hemisphere. Nutrient cycles will be affected by the precipitation changes but so far only very little is known how soil N transformations may respond. Here we investigated gross soil N transformation rates and their response to simulated rainfall events across Northeast China Transect (NECT). The results showed that gross N mineralisation rate, nitrification rate and nitrification to mineralisation ratio significantly increased as the humidity index decreased along NECT, resulting in NO_3_^−^ as the predominant inorganic N form. These characteristics could increase the risk of NO_3_^−^ losses but at the same time reduce the risk of N losses via volatilization in the semiarid and arid region. The soil-plant ecosystems have developed effective N conservation strategies in the long term with respect to the prevailing climate in arid region. However, compared to humid soils more dramatic changes of soil N transformation rates are likely to occur in arid soils, after sudden soil moisture increases. Soil N conservation mechanisms in arid regions were drastically affected when the heavy rainfall frequently occurred. Arid ecosystems are expected to be more vulnerable than humid ecosystems in response to extreme rainfall events.

In the past decades, global climate change has become more obvious. In particular, the frequency and intensity of extreme precipitation events has increased worldwide in the mid-latitude and high-latitude land areas of the Northern Hemisphere^1^,^2^,^3^. The increase in extreme precipitation events are expected to trigger more floods and droughts, which may also be affecting the soil biogeochemical cycles and increases the vulnerability of arid and semi-arid ecosystems^4^. Nitrogen (N) is a key element for all living things and also intimately linked to other biogeochemical cycles. Thus, identifying the characteristics of soil N transformation dynamics in the arid and semi-arid ecosystems and evaluating their response to extreme precipitation events can identify vulnerability triggers of ecosystems to precipitation changes. This will also provide insights for scientists and managers in developing effective adaptation and mitigation strategies.

The precipitation gradient provides a natural option to investigate the effect of a changing precipitation on N transformation dynamics. Recently, a number of researchers have investigated the effects of precipitation on the ecosystem N cycle along the natural precipitation gradient, using the natural N isotopic signature as an index to indicate the status of the ecosystem N cycle^5^,^6^,^7^,^8^,^9^,^10^,^11^. It is widely accepted that the δ^15^N values in soil and plant samples were higher in the arid regions than those in the wet regions along precipitation gradients, suggesting soil N turnover rates increased and N cycle become more ‘open’ to N loss with annual precipitation decreases^9^,^10^,^11^,^12^. However, δ^15^N values were only a qualitative estimate and any factor (e.g., aridity, tillage, extreme pH, fire, grazing, biogenic N_2_ fixation or N deposition) that can decrease N sequestration into organic matter or increase the flux from organic to mineral pools can result in the increase of δ^15^N values^7^,^9^. Therefore, the mechanisms causing the natural ^15^N enrichment in arid regions along precipitation transects are still not clear. In contrast to δ^15^N studies, results from ^15^N tracing approaches are less affected by these additional factors. Thus, gross soil N transformation rates should provide a way to confirm more directly whether soil N turnover rates become faster and the nitrogen conservation potential decreases as annual precipitation decreases^9^. Moreover, ^15^N tracing studies can provide more detailed information on N cycling processes^13^,^14^,^15^. However, up to now only few studies have been carried out to investigate the characteristics of soil gross N transformations along a precipitation transect.

We hypothesized soil N turnover rates could become faster as annual precipitation decreases the Northeast China Transect (NECT) and the arid ecosystems were expected to be more vulnerable than humid ecosystems in response to extreme rainfall events, based on the results of the previous natural N isotopic signature observations. In this study, a series of soil samples were collected along 1600 km of the NECT, covering a humidity index (HI, the ratio of precipitation to the potential evapotranspiration) gradient from 0.1 to 0.92 and the soil gross nitrogen transformations rates were measured using ^15^N tracing experiment to test our hypothesises. Our results showed that gross N mineralisation rate, nitrification rate and nitrification to mineralisation (*N/M*) ratio significantly increased as the humidity index (HI) decreased along the NECT, resulting in NO_3_^−^-N as the predominant inorganic N form. These characteristics could increase the risk of NO_3_^−^ losses but at the same time reduce the risk of N losses via volatilization in the semiarid and arid region. However, more dramatic changes of soil N transformation rates are likely to occur in arid soils compared to the humid soils, after sudden soil moisture increases. Soil N conservation mechanisms in arid regions were drastically affected when the heavy rainfall frequently occurred. These results suggest that the arid ecosystems are expected to be more vulnerable than humid ecosystems in response to extreme rainfall events.

## Results

### Soil properties

**Figure 1:**
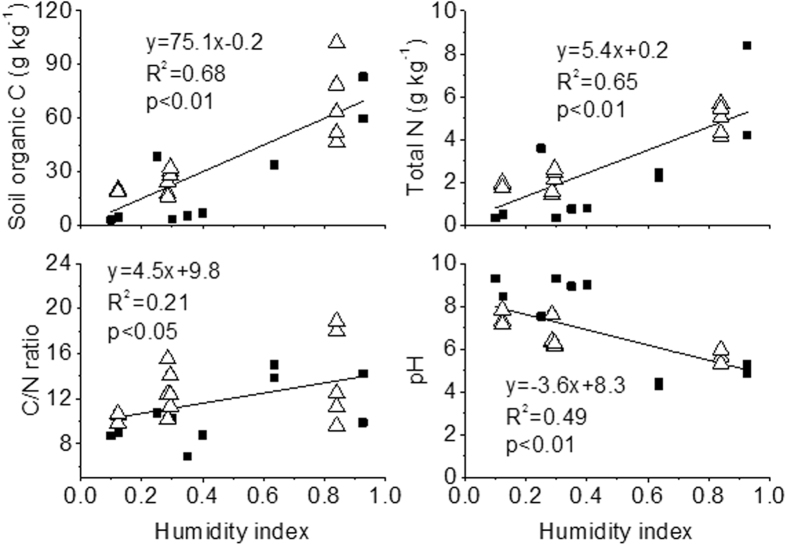
Soil properties with the increasing HI along the NECT. The hollow triangles are the data in the sample sites with spatial repetitions. The solid squares are the data measured using one composite soil sample per site.

The humidity index (HI), ranging from 0.1 to 0.92, decreased from east to west along the NECT (Fig. S2). Soil properties along the NECT were characterised by pronounced differences in pH, SOC, total N (TN) and C to N ratio (Fig. 1). Soil organic C (*P* < 0.01), TN (*P* < 0.01), and C to N ratio (*P* < 0.05) decreased, while, soil pH (*P* < 0.01) increased with the decrease in HI. Generally, soils in the wet regions were acidic, with soil pH ranging from 4.3 to 5.9, whereas soils in the semiarid and arid regions were approximately neutral and alkaline, with soil pH ranging from 6.1 to 9.3.

### The characteristics of gross N transformation along the NECT

**Figure 2:**
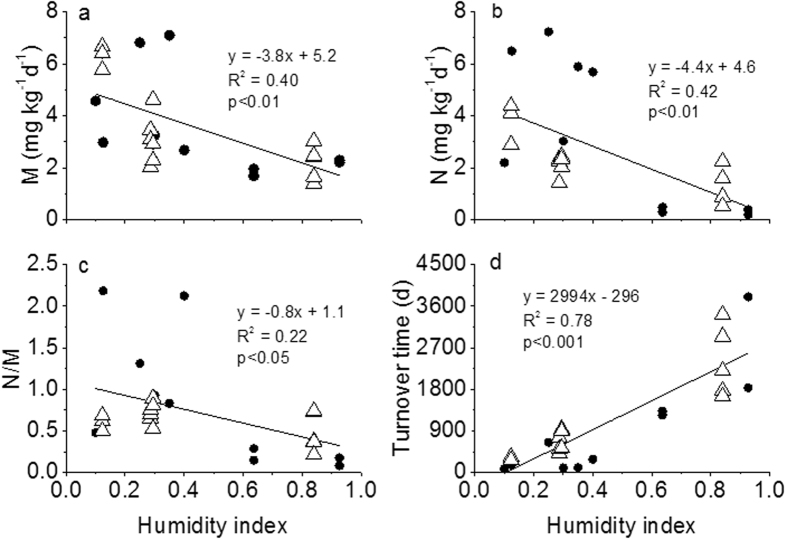
(**a**) Soil gross N transformation rates estimated by ^15^N tracer model with the increasing HI along the NECT (**c**) mineralisation rate of organic N (*M*), (**b**) autotrophic nitrification (*N*), (**c**) mineralisation to nitrification ratio (*N*/*M*), (**d**) turnover time of the organic N pool. The hollow triangles are the data in the sample sites with spatial repetitions. The solid dots are the data measured using one composite soil sample per site.

All soil gross N transformation rates were calculated using the ^15^N tracing model^15^. The gross N mineralisation rate (mineralisation of recalcitrant organic-N to NH_4_^+^ + mineralisation of labile organic-N to NH_4_^+^) increased as HI decreased (Fig. 2a) (P < 0.01). The gross N mineralisation rate was positively related to soil pH (*P* < 0.01) and negatively related to the C/N ratio (*P* < 0.01). The total organic-N pool turnover time became shorter as HI decreased (Fig. 2d), suggesting that the total N pool turnover was much faster in arid region soils than in humid region soils. The gross NH_4_^+^ immobilisation rate was positively correlated with the gross N mineralisation rate (*P* < 0.01). The net N mineralisation rates also increased as HI decreased (*P* < 0.05).

The gross autotrophic nitrification (NH_4_^+^ oxidation) rate increased as HI decreased (Fig. 2b) (*P* < 0.01). The gross autotrophic nitrification rate was positively correlated with soil pH (y = 0.56x + 5.25, R^2^ = 0.58, *P* < 0.01) and the gross N mineralisation rate (y = 0.79x−0.1678, R^2^ = 0.42, *P* < 0.01). The ratio of gross nitrification to gross mineralisation (*N*/*M*) increased with the decrease in HI (Fig. 2c) (*P* < 0.01). Thus, the NH_4_^+^ produced through mineralization could rapidly transform to NO_3_^−^ and NO_3_^−^ became the dominant inorganic N form in the arid soils (Table S1).

The rates of the heterotrophic nitrification (oxidation of recalcitrant organic-N to NO_3_^−^), the dissimilatory NO_3_^−^ reduction to NH_4_^+^ and NO_3_^−^ immobilisation were very low in all studied soils, and not significantly different along the NECT. So, the data was not shown in the paper. The net nitrification rates increased as HI decreased (*P* < 0.01).

### Response of soil N transformation to simulated rainfall events

**Figure 3:**
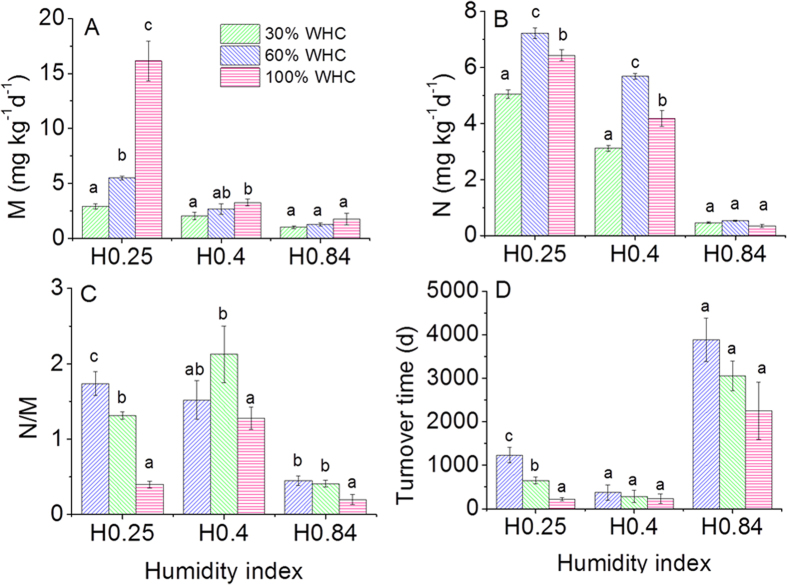
Response of soil N transformation to soil moisture change. (**A**) mineralisation rate of organic-N pool (*M*), (**B**) autotrophic nitrification (*N*), (**C**) ratio of mineralisation to nitrification (*N*/*M*), (**D**) turnover time of organic-N pool. H0.84 is soil with HI = 0.84; H0.4 is soil with HI = 0.4; H0.25 is soil with HI = 0.25. The same letter in figures indicate that the difference is not statistically significant between soil moisture treatments for same soil.

In this study, the instantaneous changes of soil moisture were used to simulate rainfall events. The response of soil N transformation to rainfall events significantly varied depending on HI in the sampled region. There were no significant response of the gross mineralization rate, gross autotrophic nitrification rate, and organic N turnover time to the instantaneous changes of soil moisture in the relatively humid region soil (HI = 0.84) (Fig. 3). However, the gross N mineralisation rate was significantly stimulated by the change of soil moisture in the arid region soil (HI = 0.25) (Fig. 3A). The gross N mineralisation rate sharply increased from 2.91 mg kg^−1^d^−1^ at 30% WHC condition and 5.51 mg kg^−1^d^−1^ at 60% WHC condition to 16.14 mg kg^−1^d^−1^ at 100% WHC condition. The highest gross autotrophic nitrification rate was observed at 60% WHC condition, and it slightly decreased at 100% WHC condition, but was still higher than that at 30% WHC condition (Fig. 3B). The *N*/*M* ratio significantly decreased with the increase in soil moisture and it was even less than 0.5 at 100% WHC condition (Fig. 3C). Moreover, the organic N turnover became much faster with the increase in soil moisture in the arid region soil (HI = 0.25) (Fig. 3D). The extent of response of soil N transformation to rainfall events in soil with HI = 0.4 fell in between HI = 0.84 and HI = 0.25. The gross N mineralisation rate and gross autotrophic nitrification rate in the arid soils at 30% WHC condition were significantly higher than those in the humid soils at 60% WHC condition (*P*<0.01).

**Figure 4:**
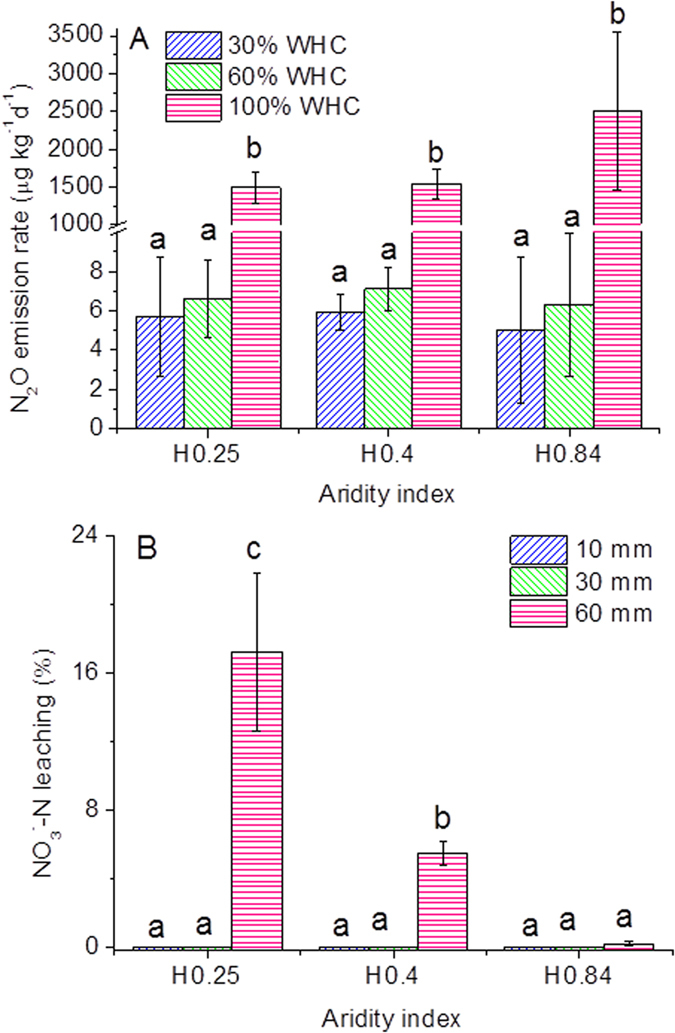
N_2_O emission (**A**) and N leaching (**B**) in the simulation of rainfall events. H0.84 is soil with HI = 0.84; H0.4 is soil with HI = 0.4; H0.25 is soil with HI = 0.25. The same letter in figures indicates that the difference is not statistically significant between soil moisture treatments for same soil.

The N_2_O emission rates were all low and were not significantly different at 30% and 60% WHC among the studied soils with different HI along the NECT (Fig. 4A). However, the N_2_O emission rate sharply increased at 100% WHC for all studied soils, compared to 30% and 60% WHC (Fig. 4A), but still did not differ significantly among the studied soils.

Soil inorganic N conservation capacity significantly varied among the studied soils with different HI along the NECT under the simulation of rainfall events (Fig. 4B). When the rainfall is low (e.g. 10 mm and 30 mm in this study), the potential leaching N loss was negligible. However, the potential leaching N loss increased with the decrease in HI in the heavy rainfall event (Fig. 4B). The amount of potential leaching N loss was still negligible, only accounting for 0.2% of applied ^15^N, in the humid region soil. Whereas, the amount of potential leaching N loss was up to 17.2% of applied ^15^N in the arid region soil.

## Discussions

Effects of precipitation changes on N transformations, as observed in this study, are in line with previous research showing that the response occurs both instantaneously and persistently^16^. The instantaneous effects on soil N transformations are in response to rainfall events and their effect on soil moisture. The persistent effects on soil N transformation refer to the response on the inherent soil properties. During soil development, climate, i.e. precipitation and temperature, is one of the key factors determining soil properties^17^, which controlled the intrinsic characteristics of soil N transformation^18^,^19^.

### The characteristics of soil N transformation along the NECT

In natural ecosystems, the characteristics of soil N cycles are often associated with conservation mechanisms in conjunction with the prevailing climate. For instance, NH_4_^+^ was generally the dominant inorganic N forms in the humid subtropical region, mainly due to low nitrification rate and low *N/M* ratio, thus reducing the risk of N loss by leaching and runoff^19^. Whereas, NO_3_^−^ was generally the predominant inorganic N forms in the arid and semiarid regions, governed by high nitrification rates, and high *N/M* ratios, thus, reducing the risk of N loss through ammonia volatilization in alkaline soils^19^,^20^,^21^. Our observation, that the gross N mineralisation rate, the nitrification rate and the *N*/*M* ratio and soil organic-N pool turnover were faster in the soils of the arid region than humid region along the NECT, is in line with previous studies^16^,^22^,^23^. The gross N mineralisation rates were positively related to soil pH (*P* <0.01) and negatively related to the C/N ratio (*P* <0.01). Since a close positive correlation existed between soil pH and HI (*P* <0.01) (Fig. 1), the correlation between N mineralisation and pH may be a persistent effect of precipitation on soil N transformation which has also been shown in several other studies^24^,^25^,^26^,^27^,^28^. The significant negative relationship between N mineralisation and the soil C/N ratio (*P* <0.01) suggested that soil organic matter composition is an important factor governing N mineralisation. Soil organic matter with lower C/N ratios are generally associated with labile SOM fractions and are more apparent in grassland compared to forest soils. Also the higher soil pH in grassland ecosystems support a faster turnover in the grassland compared to forest soils. Similar to gross mineralisation rates, the gross autotrophic nitrification rate was also positively correlated with soil pH (*P* <0.01) as also shown in other studies^19^,^29^,^30^. Moreover, autotrophic nitrification was strongly linked to total N mineralisation (*P* <0.01), which provides the NH_4_^+^ substrate for nitrification, and is another factor governing the oxidation of NH_4_^+^ to NO_3_^−^.

The *N*/*M* ratio is used to assess the capacity of autotrophic nitrifiers to compete for NH_4_^+^^31^,^32^. At a *N*/*M* ratio >0.5, NH_4_^+^ is more likely to be oxidised to NO_3_^−^. Some previous investigations also observed that the NO_3_^−^ to NH_4_^+^ ratio increased from dry sites to wet sites^20^,^21^,^33^. Compared to NH_4_^+^-N, NO_3_^−^-N was easily leached from soils in the humid region, moreover, it may rapidly denitrify under reducing conditions^34^. While, NH_4_^+^-N was able to withstand leaching in most soils, it is subject to loss through volatilization at high pH levels^35^. In the NECT, the high nitrification rate and *N*/*M* ratio results in NO_3_^−^-N being the predominant inorganic N form in soils (Table S1), reducing the risk of N loss through volatilization, in the semiarid and arid region. While, the relatively low nitrification rate and *N*/*M* ratio resulted in NH_4_^+^-N was much more dominant than NO_3_^−^-N (Table S1), successfully reducing the risk of N loss through leaching and denitrification, in the humid region. In addition, the plants’ native habitat also could determine plant N uptake preference across different ecosystems^21^,^33^,^36^,^37^. The plant species, originated from semiarid and arid environments, generally prefer to uptake NO_3_^−^-N rather than NH_4_^+^-N, because NO_3_^−^-N was the predominant inorganic N forms in soils^21^,^33^,^37^. Thus, with our study we can show that inherent connections exist in soil-plant ecosystems along the NECT with respect to effective N conservation strategies coupled to climatic drivers, despite that the N cycle appeared more ‘open’ to N loss in arid than humid regions.

### The response of soil N transformation to rainfall events along the NECT

The frequency and intensity of extreme precipitation events is increasing worldwide, especially in the mid-latitude and high-latitude land areas of the Northern Hemisphere^1^,^2^,^3^. Heavy rainfall events also increased in the west of NECT, since 1987^38^. So far it was unclear how these changes (e.g. extreme events) would impact on N transformation dynamics and possible losses arid and humid systems. It is a major challenge for us in to developing effective adaptation strategies in these regions.

Based on our results more dramatic changes in soil N transformation dynamics can be expected in arid soil regions, upon soil moisture increases from 30% WHC to 100% WHC, compared to the humid soils. Increased miscible displacement of NO_3_^−^-N may occur in these ecosystems following heavy rainfall events (60 mm) (Fig. 4B) inducing large N loss from those arid ecosystems. Despite the N_2_O emission rate was not different between the humid and arid soils, previous investigation have suggested that N_2_O was mainly produced via denitrification process in the humid forest soils, while, N_2_O only accounted for about 30% of total denitrification products in the semiarid and arid grassland soils in the NECT^39^. Thus, gaseous N loss through denitrification might be much higher in the arid soils than the humid soils (Table S1). Moreover, the organic N turnover became much faster and mineralization rate dramatically increased, producing more NH_4_^+^-N. However, at the same time, nitrification process could be relatively depressed due to high soil moisture as indicated by *N/M* ratios < 0.5. Thus, NH_4_^+^-N could temporarily become the dominant inorganic N form in these alkaline soils, which might result in larger N loss through volatilization from the arid ecosystems. Furthermore, the dominant inorganic N form (NH_4_^+^-N) in soils did not match the natural plant preference for N in form of NO_3_^−^-N^40^, even though the soil moisture condition was suitable for plant growth after the heavy rainfall events. Thus the plants competition for inorganic N with N loss could possibly been inhibited. Therefore, arid ecosystems are most likely more vulnerable than the humid ecosystems when heavy rainfall events occur more frequently.

Our results suggested that the soils in the arid ecosystems would leach more N when heavy rainfall events (60 mm) occur. However, only the top 20 cm of soil was examined and the component of plant uptake was excluded as well in the present study. When considering the likelihood that plant growth could compete for a portion of the mineralized N and the deeper soil could also retain a part of the mineralized N, the N leaching loss would likely be reduced. Therefore, the further field experiments were needed to perform to confirm the response of soil N transformations and N fate to rainfall events in the studied region.

## Materials and Methods

### Soil sampling

The study was conducted along a 1600 km stretch of the NECT across Heilongjiang, Jinlin, Liaoning province, and Inner Mongolia in northern China^41^, covering a longitude of 16° from 112°48'40" to 128°50'33" and a latitude from 43°5'22" to 47°07'11". The Northeast China Transect (NECT) is identified as a mid-latitude semi-arid terrestrial transect by the Global Change and Terrestrial Ecosystems, which is a core project of the International Geosphere-Biosphere Programme^40^. The precipitation ranges from 600–1000 mm in the east, 300–600 mm in the central region, and 100–300 mm in the west. Due to the wide range of precipitation (100–1000 mm), vegetation along the transect varies gradually from temperate evergreen conifer-deciduous broadleaf mixed forests, deciduous broadleaf forests, woodlands, and shrub lands in the east to typical steppe and desert ecosystem in the western parts^41^. Climatic conditions (steep moisture gradient) and vegetation type have affected soil properties. In 2014, 14 sites were selected throughout the transect, which were away from cities and without significant human influences. At four sites that HI is 0.84 (5 repetitions), 0.29 (3 repetitions), 0.28 (3 repetitions) and 0.12 (3 repetitions), respectively, the spatial repetitions based on the different dominant vegetation type were sampled to indicate the variability of soil N transformations within site in the NECT. At the other sites, three plots were selected. The distance between plots at each site was >1000 m. In each plot, five soil cores were taken randomly to a depth of 20 cm and mixed thoroughly into one composite sample. Thus, 24 soil samples were prepared to study soil gross N transformations. Soil samples were passed through a 2 mm sieve, and split into two subsamples, used for incubation studies and analysis of soil properties, respectively.

### ^15^N tracing experiment

The gross N transformation rates were measured in all soil samples selected from 24 sites in the NECT transect. A ^15^N tracing experiment was carried out using the incubation method of Zhang *et al*.^30^ with some modifications. Briefly, there were two NH_4_NO_3_ treatments (i.e. ^15^NH_4_NO_3_ and NH_4_^15^NO_3_, with ^15^N at 9.81, and 9.79 atom % excess, respectively). For each soil, a series of 250 ml conical flasks containing 20 g of fresh soil (oven-dry basis) were prepared. One ml of ^15^NH_4_NO_3_ or NH_4_^15^NO_3_ solution was added to each conical flask at a rate of 20 mg NH_4_^+^-N kg^−1^ soil and 20 mg NO_3_^−^-N kg^−1^ soil, respectively. The soils were adjusted to 60% water holding capacity (WHC) and incubated at 25 °C for 72 hours. The soil samples (three repetitions for each ^15^N label treatment) were extracted 0.5, 24, 48 and 72 hours after N application to determine the concentration and isotopic composition of NH_4_^+^ and NO_3_^−^.

Three soils, sampled from humid region (HI = 0.84), semiarid region (HI = 0.4), and arid region (HI = 0.25), respectively, were selected from 24 sites to study the response of soil N transformation on the rainfall events. The instantaneous changes of soil moisture were used to reflect the rainfall event. Three soil moisture treatments, 30% WHC, 60% WHC, and 100% WHC, were set up in this study. The ^15^N tracing experiment was setup according to the abovementioned procedure. In this experiment, N_2_O emission rate were also measured. Six conical flasks, three labelled ^15^NH_4_NO_3_ and three labelled NH_4_^15^NO_3_, respectively, were prepared and gas samples (30 mL) were collected from each Erlenmeyer flasks to determine N_2_O concentration (Agilent 7890A gas chromatogram). Before sampling, the flasks were flushed with ambient air for about 30 min and resealed with stoppers for 4 h.

### The potential N leaching experiment

The same three soils, sampled from humid region (HI = 0.84), semiarid region (HI = 0.4), and arid region (HI = 0.25), were selected to study the potential N leaching when the heavy rainfall events occurred. Fresh soil (200 g dry weight) was packed into polyvinyl chloride cylinder cores (5.0 cm diameter × 15 cm length) with a bulk density corresponding to the natural conditions The depth of soil layer was about 10 cm. The ^15^N labelled (NH_4_)_2_SO_4_ solution (at 9.81 atom % excess) was uniformly applied to soil core using injections at a rate of 20 mg N kg^−1^ dry soil, and incubated at 40% WHC and 25 °C for 2 days. After that, the 10, 30, 60 mm rainfall was simulated through adding distilled water to the top of soil core and the leaching was executed following 6.5 h equilibrium. Leachate was collected through the valve fitted in the bottom of PVC cylinder for about 2 h, until no liquid dropped. After that, soil in cores was also sampled and was extracted using KCl solution. The concentration and isotopic composition of NH_4_^+^ and NO_3_^−^ in leachate and in KCl extraction, and soil organic N concentration and isotopic composition were measured.

### Analysis

Soil properties (e.g. organic carbon [SOC], total N, pH, initial mineral N [NH_4_^+^ and NO_3_^−^] concentrations) were determined following the Soil Agro-Chemical Analysis procedures of Lu (2000)^42^. The isotopic composition of NH_4_^+^ and NO_3_^−^ was determined using an automated carbon/nitrogen (C/N) analyser coupled to an isotope ratio mass spectrometer (IRMS 20–22, SerCon, Crewe, UK). For detailed descriptions see Zhang *et al*.^30^.

### ^15^N tracing model setup

In the present investigation, the combination of a ^15^N tracing experiment and a process-based N cycle model was used to quantify simultaneously occurring gross N transformation rates in soil^15^. Briefly, ten simultaneously occurring gross N transformations in soil were quantified with a process-based ^15^N tracing model (Fig. S1). The transformation rates were calculated using zero-, first-order or Michaelis-Menten kinetics. Based on the kinetic settings and the final parameters, average N transformation rates were calculated over the whole period and expressed in units of mg N kg^−1^ soil day^−1^. The detailed descriptions of the ^15^N tracing model are in Müller *et al*.^15^ and Zhang *et al*.^30^. The total N turnover rate was calculated using the gross mineralization rate divided by total soil N^14^.

### Statistical analyses

Pearson correlation and multiple regression analyses were performed to explore the relationship between gross N transformation rates and soil variables (i.e. organic C, total N, ratio of C to N, pH, HI). Differences in gross N transformations, N leaching, N_2_O emission among different treatments were assessed using one-way ANOVA followed by Tukey HSD tests. All statistics were carried out with the SPSS software package 13.0 for Windows.

## Additional Information

**How to cite this article**: Zhang, J.- *et al*. Soil gross nitrogen transformations along the Northeast China Transect (NECT) and their response to simulated rainfall events. *Sci. Rep.* **6**, 22830; doi: 10.1038/srep22830 (2016).

## Supplementary Material

Supplementary Information
