# Supplementary material for: Soil gross nitrogen transformations along the Northeast China Transect (NECT) and their response to simulated rainfall events
Source: Sci Rep. 2016 Mar 7;6:22830. doi: 10.1038/srep22830 (PMC4780001; doi:10.1038/srep22830)
Supplement: Supplementary Information [file srep22830-s1.pdf]

Title: Soil gross nitrogen transformations along the Northeast China Transect (NECT) and their response to simulated rainfall events

Authors: Jin-bo Zhang[1,2,3,4], Liang Wang[5], Wei Zhao[1], Hui-feng Hu[5], Xiao-juan Feng[5], Christoph Müller[6,7], Zu-cong Cai[1,2,3,4*]

Affiliations:

1 School of Geography Sciences, Nanjing Normal University, Nanjing 210023, China

2 Jiangsu Center for Collaborative Innovation in Geographical Information Resource Development and Application, Nanjing 210023, China

3 Key Laboratory of Vitual Geographical Environment(VGE), Ministry of Education, Nanjing Normal University, Nanjing 210023, China

4 Jiangsu Provincial Key Laboratory of Materials Cycling and Pollution Control, Nanjing 210023, China

5 Institute of Botany, the Chinese Academy of Sciences, Beijing 100093, China

6 Department of Plant Ecology (IFZ), Justus-Liebig University Giessen, Heinrich-Buff-Ring 26, 35392 Giessen, Germany

7 School of Biology and Environmental Science, University College Dublin, Belfield, Dublin 4

Author contributions

All authors worked out the study aims, discussed the results and edited/commented on the manuscript. JINBO ZHANG, LIANG WANG and HUIFENG HU participated in field sampling campaigns; JINBO ZHANG and WEI ZHAO prepared experimental set-up and scientific protocols. JINBO ZHANG wrote the manuscript, and carried out data analysis; XIAOJUAN FENG, ZUCONG CAI and CHRISTOPH MÜLLER supervised the project.

Competing financial interests

The authors declare no competing financial interests

[*]**Corresponding author**: Zucong Cai

Address: School of Geography Sciences, Nanjing Normal University, Nanjing 210023, China

Tel.: +86 25 8589 1203, Fax: +86 25 8589 1745;

E-mail addresses: zccai@njnu.edu.cn (Zucong Cai)

Type of articles: ***article***

Number of words in the introductory paragraph: 200

Number of words in the main text: 3552

Number of references: 42

Number of figures, and tables: 4

Running title: Soil gross N transformations along the NECT

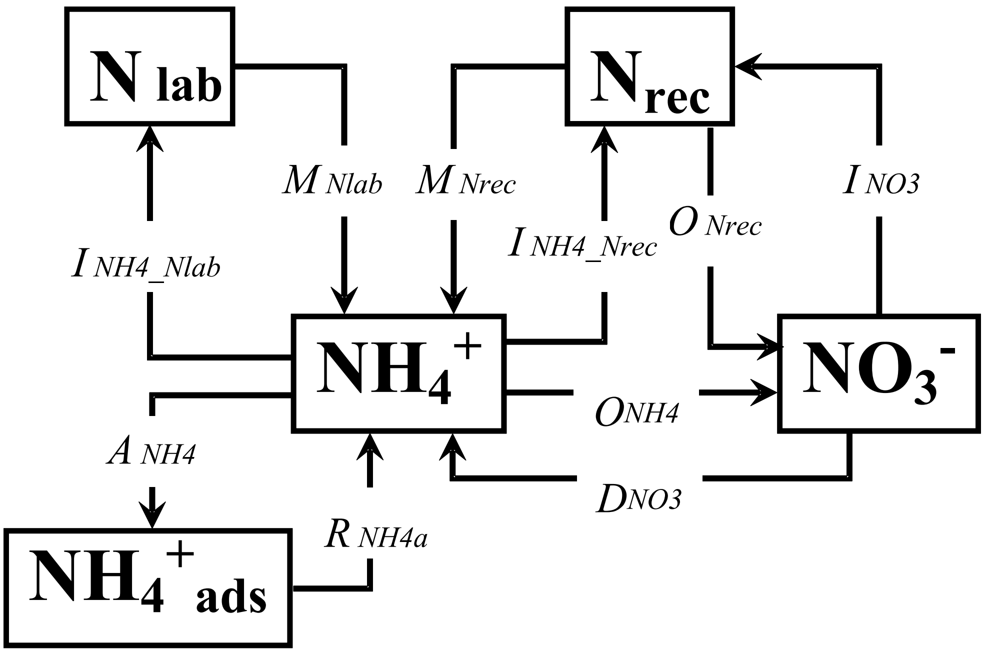

Figure S1. [15]N tracing model used for data analysis in the present study. The model is from Müller et al. (2007). N_{lab} = labile soil organic-N, N_{rec} = recalcitrant soil organic-N, NH_4^+ = ammonium, NO_3^- = nitrate, $NH_4^+{}_{\mathrm{ads}}$ = adsorbed NH_4^+; M_{Nrec} = mineralisation of recalcitrant organic-N to NH_4^+; M_{Nlab} = mineralisation of labile organic-N to NH_4^+; $I_{\mathrm{NH4\_Nlab}}$ = immobilisation of NH_4^+ to labile organic-N; $I_{\mathrm{NH4\_Nrec}}$ = immobilisation of NH_4^+ to recalcitrant organic-N; R_{NH4ads} = release of adsorbed NH_4^+; A_{NH4} = adsorption of NH_4^+ on cation exchange sites; O_{NH4} = oxidation of NH_4^+ to NO_3^-; O_{Nrec} = oxidation of recalcitrant organic-N to NO_3^-; I_{NO3} = immobilisation of NO_3^- to recalcitrant organic-N; D_{NO3} = dissimilatory NO_3^- reduction to NH_4^+.

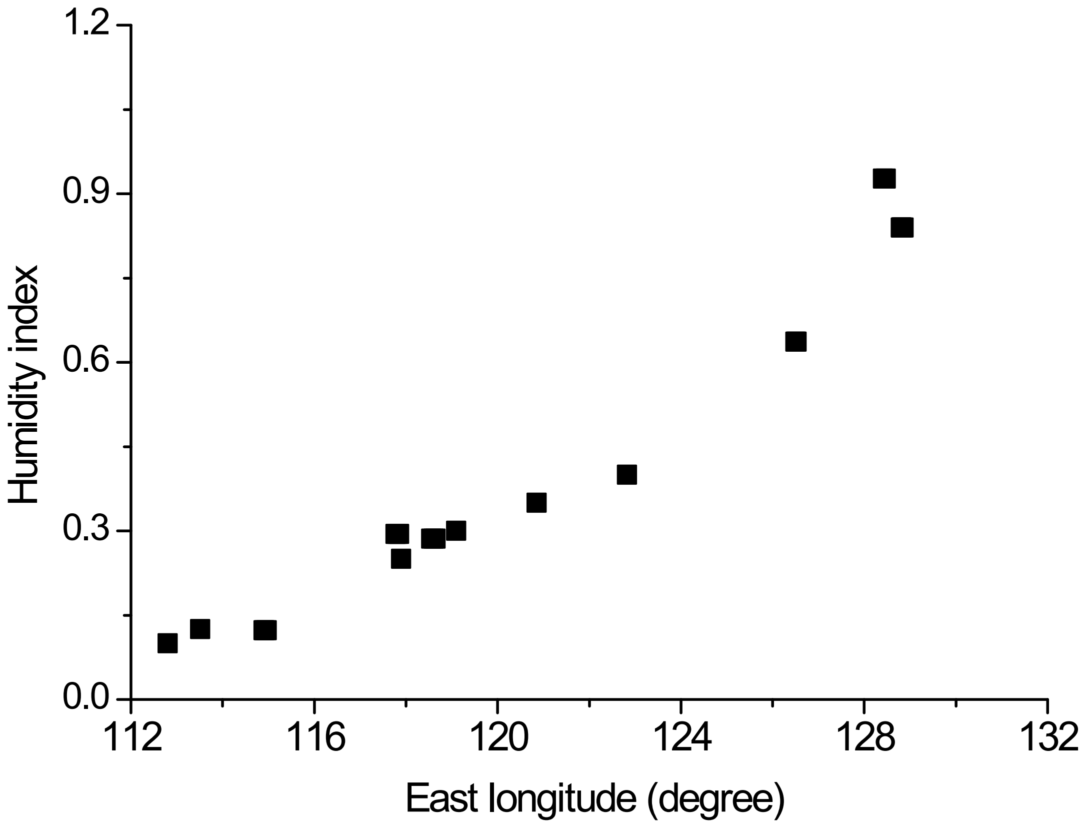

Figure S2 The change of aridity index in the sample sites along the NECT in this study

Table S1 The fate of labeled ^{15}N-NH_4^+ under simulated rainfall conditions in soils developed under different aridity index

Aridity index	Simulated rainfall	NH_4^+-N	NO_3^--N	SON	Leaching N %		Unrecovered N
	mm	%	%	%	NH_4^+-N	NO_3^--N	%
1.2	10	81.5±5.9	2.9±1.0	13.1±1.4	0	0	2.5
	30	79.1±5.3	3.3±0.4	15.2±2.9	0	0	2.4
	60	77.4±2.1	2.0±0.3	16.5±1.5	0.1±0.1	0.2±0.1	3.8
2.5	10	28.6±2.5	58.8±2.7	10.8±1.8	0	0	1.8
	30	27.8±16.7	64.1±1.0	11.3±4.5	0	0	-3.2
	60	24.8±11.4	46.3±1.1	14.5±2.5	0	5.5±0.7	8.9
4	10	11.9±6.6	65.1±7.8	20.1±4.7	0	0	2.9
	30	10.3±1.7	63.2±2.8	22.6±5.1	0	0	3.9
	60	11.8±2.9	44.3±2.9	17.1±0.9	0	17.2±4.6	9.6
